# Hepcidin and Iron in Health and Disease

**DOI:** 10.1146/annurev-med-043021-032816

**Published:** 2022-07-29

**Authors:** Elizabeta Nemeth, Tomas Ganz

**Affiliations:** Center for Iron Disorders, Department of Medicine, David Geffen School of Medicine, University of California, Los Angeles, California, USA

**Keywords:** hepcidin, iron, inflammation, anemia, iron overload

## Abstract

Hepcidin, the iron-regulatory hormone, determines plasma iron concentrations and total body iron content. Hepcidin, secreted by hepatocytes, functions by controlling the activity of the cellular iron exporter ferroportin, which delivers iron to plasma from intestinal iron absorption and from iron stores. Hepcidin concentration in plasma is increased by iron loading and inflammation and is suppressed by erythropoietic stimulation and during pregnancy. Hepcidin deficiency causes iron overload in hemochromatosis and anemias with ineffective erythropoiesis. Hepcidin excess causes iron-restrictive anemias including anemia of inflammation. The development of hepcidin diagnostics and therapeutic agonists and antagonists should improve the treatment of iron disorders.

## SYSTEMIC IRON HOMEOSTASIS

Iron is an essential nutrient and a component of ferroproteins and enzymes that carry out vital biochemical functions. Despite being one of the most abundant elements in our planet, the commonly occurring forms of iron are insoluble and poorly bioavailable in many diets. Normally, humans conserve iron very efficiently, losing less than 0.1% of their body iron content each day, an amount that is replaced through dietary iron absorption. Most of the iron in the body is in the hemoglobin of red cells, which contain approximately 1 mg per milliliter of cells or approximately 2–2.5 g total. Blood loss in excess of dietary iron absorption is the main cause of iron deficiency worldwide.

The concentration of iron in plasma is maintained in the range of 10–30 μM, and long-term deviations from this range result in disease. Decreased hemoglobin synthesis and anemia are the most common consequences of low plasma iron concentrations (hypoferremia). Severe hypoferremia can also compromise the synthesis of ferroproteins in nonerythroid cell types, causing cellular dysfunction and additional manifestations of iron deficiency, including epithelial changes in nails, tongue, and esophagus; deficits in cognitive and muscle performance; and impaired adaptive immunity responses (1). At the other extreme, high plasma iron concentrations can exceed the ironbinding capacity of transferrin and generate complexes with other plasma proteins as well as with organic anions such as citrate. This non-transferrin-bound iron (NTBI) is avidly taken up by hepatocytes and other parenchymal cells through transporters that, under physiological conditions, primarily transport other metals. Rapid and excessive accumulation of intracellular iron causes cell and tissue damage, presumably by iron-catalyzed generation of reactive oxygen species (2).

## THE ROLE OF THE HEPCIDIN–FERROPORTIN AXIS IN IRON HOMEOSTASIS

### Overview of Hepcidin Functions

**Figure 1:**
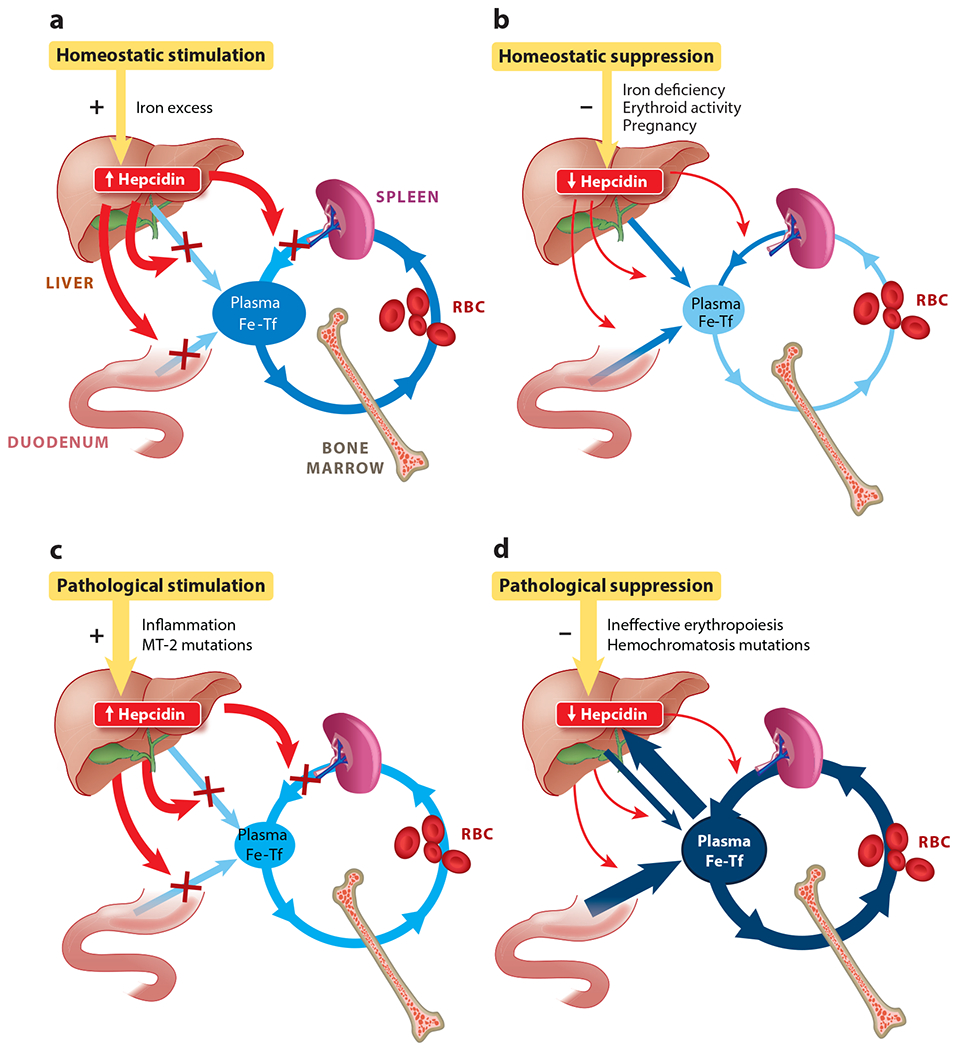
Hepcidin has a homeostatic role in conditions associated with physiological hepcidin stimulation (*a*) or suppression (*b*) and a pathological role in iron-restrictive disorders (*c*) and iron-overload disorders (*d*). Iron flows are shown in shades of blue indicative of intensity. Hepcidin and its effects are shown in red; normal and pathological hepcidin modulators are shown in gold. Abbreviations: Fe-Tf, iron-transferrin; MT-2, matriptase-2; RBC, red blood cell. Figure adapted from Reference 5 with permission.

Hepcidin is a 25-amino-acid peptide hormone secreted by hepatocytes (3). Hepcidin negatively regulates the main iron flows that enter the plasma compartment (Figure 1): the absorption of dietary iron in the duodenum, the release of recycled iron from macrophages, and the release of stored iron from hepatocytes (4). Iron and hepcidin regulate each other through a classical endocrine feedback loop. When iron is abundant, more hepcidin is produced by hepatocytes, limiting further iron absorption and release of iron from stores (Figure 1a). When iron is deficient, hepatocytes produce less hepcidin, allowing more iron to enter plasma (Figure 1b). The specific forms of iron that increase hepcidin synthesis include diferric plasma transferrin and stored iron in hepatocytes.

In addition to the regulation by hepatic and extracellular iron, hepcidin is also homeostatically regulated by the erythropoietic need for iron, as well as during pregnancy to meet the increased iron requirements of the mother and the fetus (Figure 1b). During active erythropoiesis, hepcidin production is suppressed, thereby increasing iron availability for hemoglobin synthesis (6). The principal erythropoietic suppressor of hepcidin (erythroid factor) is erythroferrone, a hormone secreted by erythropoietin-stimulated erythroblasts and acting on the liver to decrease hepcidin production (see below). During the second and third trimesters of pregnancy, hepcidin is profoundly suppressed, allowing increased mobilization of iron for fetal development and the expansion of maternal erythrocyte numbers (7). The suppressive signal likely originates in the placenta but has not yet been molecularly characterized.

Another function of hepcidin is as a mediator of innate immunity, consistent with its likely evolutionary origin as an antimicrobial peptide. During infections and other inflammatory events, the production of hepcidin is stimulated by interleukin (IL)-6 and other cytokines (8). Increased hepcidin concentrations cause the retention of iron within macrophages, counteracting the otherwise increased recycling of iron from damaged tissues during infection. Slowing the entry of iron into plasma and extracellular fluid allows transferrin to sweep up any NTBI, an iron species that is highly bioavailable to gram-negative bacteria and other microbes and stimulates their rapid growth (9).

Hepatocytes are the main source of circulating hepcidin, which regulates systemic iron homeostasis. However, cell types such as cardiomyocytes, dendritic cells, and keratinocytes also express hepcidin mRNA, and these sources of hepcidin have an autocrine and paracrine effect on local iron distribution. Cardiomyocyte hepcidin appears to maintain baseline iron homeostasis of the heart (10), and keratinocyte and dendritic cell hepcidin facilitates the local response to infection and inflammation (11, 12).

## IRON DELIVERY TO PLASMA IS DEPENDENT ON THE CELLULAR IRON EXPORTER FERROPORTIN

Iron absorbed from the diet by intestinal enterocytes, recycled by macrophages, or stored in hepatocytes is transported across their cell membranes into the plasma via the iron exporter ferroportin (13). Ferroportin is a 12-transmembrane domain protein and the only conduit for elemental iron entry into the plasma. Ferroportin does not bear any significant structural similarities to other membrane transport proteins, but it has been highly conserved during evolution and is found in plants, invertebrates, and even some microbial species (14). In humans, ferroportin is found not only in enterocytes, macrophages, and hepatocytes, which handle large quantities of iron, but also in erythrocyte precursors in the bone marrow and even in mature erythrocytes, where it may function to return to plasma any extra iron that may otherwise be toxic to these cells (15). Ferroportin expression on most other cell types is minimal and contributes little to systemic iron homeostasis but may be important for local iron homeostasis and detoxification of reactive iron.

### Hepcidin Inactivates Ferroportin to Decrease Iron Transfer to Plasma

Hepcidin inhibits cellular iron efflux not only by directly binding to and occluding ferroportin (16) but also by inducing a conformational change in ferroportin that results in ferroportin ubiquitination (17), endocytosis of both molecules, and their lysosomal degradation (18). In vivo, the loss of iron efflux into plasma from macrophages, hepatocytes, and enterocytes results in hypoferremia because of continued consumption of plasma iron, predominantly for hemoglobin synthesis by erythrocyte precursors. The structural basis of hepcidin–ferroportin interaction is becoming clearer as a result of detailed structure–function studies and recent cryoelectron microscopic analysis of the hepcidin–ferroportin complex (19). The N-terminal 9-amino-acid segment of hepcidin is necessary and sufficient for the hepcidin–ferroportin interaction, and it has served as a template for the development of minihepcidins and other small drug-like hepcidin agonists (20).

### Regulation of Iron Absorption by Hepcidin

During iron deficiency, low hepcidin levels result in greater ferroportin activity on duodenal enterocytes, leading to the depletion of enterocyte iron levels. The lack of enterocyte iron decreases the activity of oxygen- and iron-dependent prolyl hydroxylases that target hypoxia-inducible factors (HIFs) for degradation in proteasomes, and results in stabilization of the HIFs. HIF2α is an important local regulator of transcription of the apical iron importer DMT1 (divalent metal transporter 1), the iron reductase DCYTB (duodenal cytochrome *b*), and the basolateral exporter ferroportin (21). Stabilization of HIF2α thus coordinates apical import of dietary iron with the hepcidin-controlled activity of ferroportin and results in enhanced absorption of intestinal iron during conditions of iron deficiency. The same mechanism contributes to pathologically increased iron absorption in conditions associated with hepcidin deficiency (most commonly hemochromatosis and iron-loading anemias).

## PATHOPHYSIOLOGY OF HEPCIDIN REGULATION

### Iron Regulates Hepcidin Transcription in Hepatocytes

**Figure 2:**
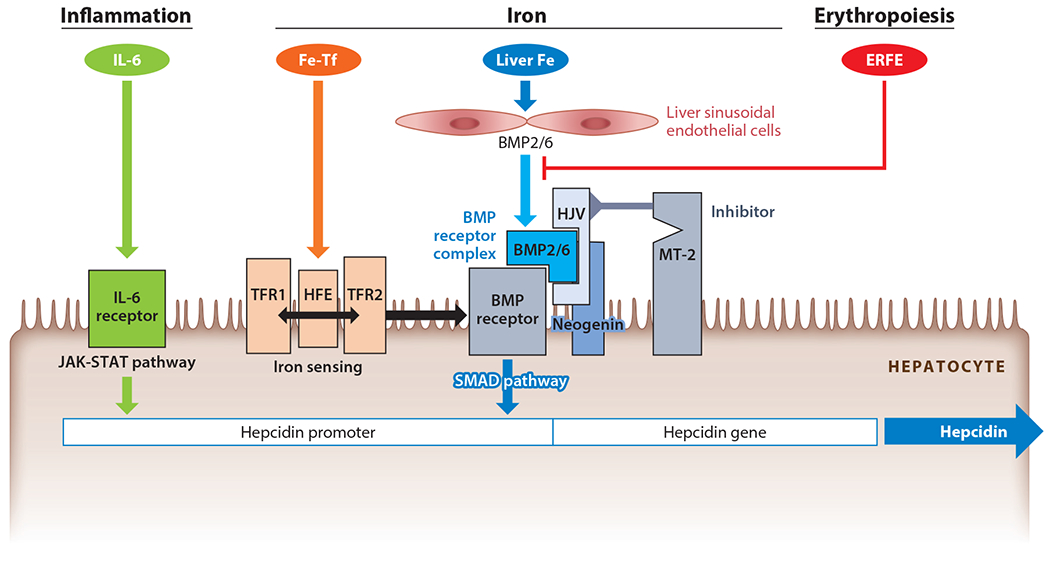
Molecular mechanisms of hepcidin regulation. *Center*: Iron regulates hepcidin through two distinct mechanisms. Extracellular iron in the form of holotransferrin (Fe-Tf) is sensed by the two transferrin receptors (TFR1 and TFR2). Binding of Fe-Tf to its receptors promotes hemochromatosis protein (HFE) interaction with TFR2 instead of TFR1, and the HFE/TFR2 complex then sensitizes the bone morphogenetic protein (BMP) receptor to its ligands BMP2 and −6 or the BMP2/6 heterodimer. HFE may also directly stabilize the BMP receptor by preventing its ubiquitination. Hemojuvelin (HJV), a membrane-linked BMP coreceptor, potentiates the BMP receptor activation. Once activated, BMP receptors initiate SMAD signaling, which increases hepcidin transcription. Increased intracellular iron in the liver enhances BMP6 and −2 production by liver sinusoidal endothelial cells, eventually leading to activation of the BMP receptor on hepatocytes. Under low-iron conditions, low Fe-Tf concentrations and low intracellular iron both lead to decreased BMP pathway signaling and decreased hepcidin mRNA expression. Furthermore, matriptase-2 (MT-2) protease is stabilized in low-iron conditions and inhibits and cleaves HJV and other molecules of the BMP pathway, thus further decreasing the SMAD signaling. *Left*: Inflammation stimulates hepcidin production by increasing the transcription of hepcidin through the interleukin (IL)-6–JAK-STAT pathway. *Right*: Erythropoiesis activation leads to increased production of erythroferrone (ERFE) by erythroblasts. ERFE then inhibits BMP signaling by binding to BMP2/6 and interfering with its interaction with the BMP receptor, thereby lowering hepcidin and making more iron available for erythropoiesis.

Like other hormones, hepcidin is feedback-regulated by the substance whose concentration it controls, iron. Both extracellular iron, in the form of iron-transferrin, and intracellular hepatic iron regulate hepcidin synthesis, although the mechanism of intracellular iron sensing is still unknown. Both sensing mechanisms converge onto the bone morphogenetic protein (BMP) pathway to increase hepcidin transcription (Figure 2). The BMP pathway regulates many processes, including embryonic morphogenesis, bone development and remodeling, and tissue repair. In hepatocytes, the BMP receptors ALK2 and −3, as well as ligands BMP2 and −6, were shown to specifically regulate hepcidin, together with a membrane-anchored coreceptor, hemojuvelin (22). Binding of the BMP ligands to the receptors activates SMAD 1/5/8 phosphorylation and increases hepcidin transcription. The BMP pathway activity sets the hepcidin baseline and is further modulated by iron concentrations (23). Two transferrin receptors, TFR1 and TFR2, along with the membrane protein HFE (which interacts with both receptors), function as sensors of extracellular iron (diferric transferrin) (Figure 2). HFE interaction with TFR1 is modulated by holotransferrin: As HFE and holotransferrin bind to overlapping sites on TFR1, increasing concentrations of holotransferrin displace HFE from its association with TFR1. HFE is then thought to interact with the BMP receptor ALK3 and stabilize it, thus potentiating BMP signaling. Binding of holotransferrin to TFR2 prevents TFR2 degradation and may also promote TFR2 interaction with the BMP receptors. The structural interactions that allow the iron sensors to drive BMP signaling and hepcidin transcription remain to be elucidated.

At least two different cell types in the liver are required for normal hepcidin regulation (22). The sinusoidal endothelial cells function as a source of BMP2 and BMP6, which are required for hepcidin regulation in hepatocytes located across the narrow space of Disse (24, 25) (Figure 2). In addition to the response to holotransferrin, hepatocytes can also increase hepcidin synthesis in response to stored intracellular iron. The mechanisms are not known, but BMP6 expression was shown to positively correlate with iron loading (26), suggesting that BMP6 concentrations could reflect the hepatocyte or sinusoidal endothelial cell intracellular iron levels.

### Erythropoietic Activity Suppresses Hepcidin Synthesis Through Erythroferrone

Erythropoietic precursors in the bone marrow are the main consumers of iron from holotransferrin. Appropriately, the expansion of the erythroid precursor population, such as after bleeding or administration of erythropoietin, suppresses hepcidin through a mediator released by the bone marrow. Studies during the last decade have identified erythroferrone as a key erythroid factor mediating the suppression of hepcidin in response to bleeding or exogenous erythropoietin (5). The homeostatic mechanism can become pathological in diseases with ineffective erythropoiesis, where erythrocyte precursors massively expand but undergo apoptosis rather than maturing into erythrocytes. This mechanism, partially mediated by erythroferrone, is responsible for low hepcidin in β-thalassemia (27).

Accumulating evidence indicates that erythroferrone suppresses hepcidin transcription by interfering with BMP signaling, presumably in the space of Disse where erythroferrone could interact with BMP2 and −6 and interfere with their access to BMP receptors (28) (Figure 2). However, other mechanisms could also contribute to hepcidin suppression after erythropoietic stimulation. One such alternative mechanism is erythropoietin-dependent increased TFR1 expression on erythroblasts, causing rapid depletion from plasma of its preferred ligand, diferric transferrin (29). As diferric transferrin also stimulates hepcidin transcription through transferrin receptors TFR1 and TFR2 on hepatocytes, depletion of diferric transferrin would be expected to lower hepcidin secretion by decreasing SMAD signaling.

### Hepcidin Suppression During Pregnancy Is Essential for Normal Embryonic Development

During the second and third trimesters of human pregnancy, large amounts of iron must be mobilized to support fetal growth and erythropoiesis and to expand maternal erythrocytes. This appears to be achieved through physiological suppression of hepcidin production, probably by an as-yet-uncharacterized placental hormone. Inappropriately increased maternal hepcidin activity in mouse pregnancy causes adverse fetal outcomes, including iron-deficiency anemia in embryos and decreased embryo weight (7). In human pregnancy, increased maternal hepcidin was associated with very low birth weight with an odds ratio of 3 (30).

### Inflammation Increases Hepcidin Synthesis Through IL-6 and Other Mediators

Hepcidin production increases during infections and systemic inflammatory diseases, reflecting the role of hepcidin as a mediator of innate immunity. In this context, hepcidin synthesis by hepatocytes is transcriptionally regulated by IL-6 through the STAT-3 signaling pathway; BMP signaling synergizes with IL-6 to increase hepcidin (8, 31) (Figure 2). Elevated hepcidin is responsible for the hypoferremia that develops early during infections or in inflammatory diseases (Figure 1c). The inflammatory hepcidin response likely evolved to prevent generation of NTBI during infection (9), when the risk of transferrin oversaturation is higher because erythropoiesis is inhibited (decreasing consumption of plasma iron) and recycling of iron from damaged cells by macrophages increases (32). Thus, the role of hepcidin in host defense is to block the production of NTBI to prevent rapid growth of NTBI-dependent extracellular microbes. However, iron sequestration and hypoferremia due to inflammation-induced hepcidin also limit the availability of iron for erythropoiesis and contribute to anemia of inflammation (also known as anemia of chronic disease) (33).

### Impaired Renal Clearance of Hepcidin Leads to Its Accumulation

Due to its small size (2.7 kD) and modest binding to other plasma proteins, hepcidin readily passes through the glomerular membrane and, like other small proteins, is then taken up and degraded in the proximal tubule (34). A small fraction of the filtered hepcidin passes intact into urine, where it is readily detectable and correlates with circulating hepcidin levels. Chronic kidney disease (CKD) impairs the clearance of hepcidin, leading to its accumulation in plasma, where it may contribute to iron sequestration in macrophages and limit the availability of iron for erythropoiesis. Along with increased inflammation and impaired production of erythropoietin, this mechanism may contribute significantly to anemia of CKD. Hepcidin is efficiently cleared during hemodialysis, but its high rate of production restores predialysis concentrations within hours (35).

## IRON OVERLOAD DISORDERS DUE TO HEPCIDIN DEFICIENCY OR RESISTANCE TO HEPCIDIN

### Hemochromatosis

Hemochromatosis (also called hereditary hemochromatosis) is a group of primary genetic disorders of iron homeostasis in which hyperabsorption of dietary iron leads to iron accumulation in tissues, resulting in iron-mediated injury and organ dysfunction. Iron accumulates because humans lack compensatory mechanisms that would significantly increase iron excretion in response to iron excess. Although the absorption of iron takes place in the duodenum, mouse models have demonstrated that the primary locus of the disorder is hepatocytes that fail to produce a sufficient amount of hepcidin to effectively regulate iron absorption in the duodenum (36). The severity of the hepcidin impairment determines the rate of progression of iron overload and the clinical course. Inadequate hepcidin production can be caused by autosomal recessive mutations in the hepcidin gene itself or in genes encoding hepcidin regulators HFE (common), TFR2, or hemojuvelin (a membrane-anchored coreceptor) (Figure 1d). When hepcidin is deficient, the iron exporter ferroportin is overexpressed on the basolateral membranes of duodenal enterocytes, increasing dietary iron absorption. Macrophages also overexpress ferroportin and avidly export iron, leading to relative depletion of macrophage intracellular iron. Greater influx of iron into plasma raises plasma iron concentration, saturating transferrin with iron and generating NTBI. NTBI is rapidly taken up by hepatocytes, cardiac myocytes, and endocrine cells, causing tissue damage and organ failure (37). The liver is the primary organ affected by iron overload. This is likely a consequence of the hepatocytes’ avid uptake of NTBI, exceeding any iron export from these cells and resulting in the net accumulation of excess iron. Rarely, hemochromatosis can arise from ferroportin mutations that interfere with either hepcidin binding or the resulting conformational changes, manifesting as resistance to the effects of hepcidin (16).

In the severe (“juvenile”) forms of the disease, usually due to mutations in the hepcidin gene or the hemojuvelin gene, little or no hepcidin is detectable in plasma. Juvenile hemochromatosis is highly penetrant and affects both genders equally. Clinical problems develop in late childhood or early adulthood, including endocrinopathies and cardiomyopathy. It is remarkable that the heart failure associated with hemochromatosis is usually reversible by aggressive deironing, including the use of iron chelators (38). In the adult forms of the disease, hepcidin synthesis is partially responsive to iron loading, but serum hepcidin concentrations are too low relative to iron load (39). This means that hepcidin levels may be in the normal range at the time of diagnosis but that the level is inappropriately low considering that patients are iron-overloaded. As iron is lowered to normal levels by venesections, frank hepcidin deficiency becomes manifest. The adult forms of the disease include a rare form, due to autosomal recessive mutations in the TFR2 gene, and the most common form, due to autosomal recessive mutations in the HFE gene, but digenic inheritance also occurs (40). HFE mutations are highly prevalent in populations of northern European ancestry, but the disease is incompletely penetrant and affects men more frequently than women. Most genetically affected individuals are identified because of laboratory abnormalities or family history rather than overt disease. Factors such as alcohol intake, other modulating genes, and possibly the heme content of the diet may codetermine the rate of progression (40). If untreated, a small percentage of affected individuals will develop cirrhosis, sometimes progressing to liver cancer.

Currently, hemochromatosis is treated by iron depletion through phlebotomy, with each unit of whole blood (~500 mL) removing approximately 200–250 mg of iron. Although effective, this treatment eventually leads to severe hepcidin deficiency, increasing iron absorption, and the need for additional therapeutic phlebotomies. Furthermore, phlebotomy is not suitable for all patients owing to poor vascular access, adverse physiological responses to phlebotomy, the inconvenience of travel to phlebotomy centers, and other reasons. Therapeutic correction of hepcidin deficiency may offer additional treatment options for patients suffering from hemochromatosis.

### Iron-Loading Anemias

A severe iron overload disorder develops in most patients with β-thalassemia with or without transfusion dependency, as well as in patients with other erythrocyte disorders characterized by ineffective erythropoiesis (pyruvate kinase deficiency, congenital dyserythropoietic anemias, some sideroblastic anemias) (41). In β-thalassemia, anemia drives erythropoietin production, which in turn causes massive expansion of erythroid precursors in the marrow and elsewhere. Stimulated by erythropoietin, the expanded erythron secretes high amounts of erythroferrone leading to hepcidin suppression, although other mechanisms may also contribute (42). As in hemochromatosis, hepcidin deficiency results in hyperabsorption of dietary iron and development of iron overload, even in the absence of transfusions (Figure 1d). Unless effectively treated by iron chelators, iron overload is the major cause of severe morbidity and mortality in this disease.

In β-thalassemia patients treated with regular transfusions, iron overload is primarily the consequence of the treatment, and hepcidin levels are normal or even increased, although still deficient considering the iron overload, particularly toward the end of the transfusion cycle (43). Transfusions partially correct the anemia, acutely and chronically decreasing erythropoietin secretion. The stimulus to erythroid expansion is diminished, as is the production of hepcidin-suppressive erythroferrone, thus allowing hepcidin levels to rise. Circulating hepcidin concentrations are thought to affect the distribution of iron between the macrophage storage compartment (favored by higher hepcidin concentrations) and parenchymal cells, including cardiac myocytes and hepatocytes (favored by low hepcidin) (44). The balance of iron between these two compartments may have implications for the rate of progression of cardiomyopathy. Other iron-loading anemias display similar iron pathology but are much less common.

Treatment with iron chelators can have serious adverse effects, and patient compliance is frequently a problem. In the future, treatment with hepcidin agonists may become an alternative or addition to chelation therapy, at least in untransfused patients.

## IRON-RESTRICTIVE DISORDERS DUE TO HEPCIDIN EXCESS OR FERROPORTIN DEFICIENCY

The term iron restriction refers to a condition where hypoferremia limits delivery of iron to consuming tissues, especially the erythropoietic marrow, despite adequate or even increased total body iron. Unlike iron deficiency, which denotes decreased total amount of iron in the organism, iron restriction implies maldistribution of iron, caused by sequestration of iron in macrophages.

### Iron-Refractory Iron Deficiency Anemia

Pure hepcidin excess, even in the absence of inflammation, causes hypoferremia and microcytic anemia. This occurs in familial iron-refractory iron deficiency anemia (IRIDA), an autosomal recessive disorder caused by mutations in a negative regulator of hepcidin, the membrane serine protease matriptase-2 (also called TMPRSS6) (45) (Figure 2). Despite severe iron deficiency, which would be expected to physiologically suppress hepcidin production, patients with IRIDA show high-normal or even increased serum hepcidin (46) (Figure 1c). Matriptase-2 has been shown to cleave membrane hemojuvelin and other components of the BMP receptor hepcidin-regulatory complex (47). Recent studies indicate that matriptase-2 also has a nonenzymatic inhibitor function dependent on its conformational properties (48), perhaps explaining the broad distribution of pathogenic mutations in the protein. Because of elevated hepcidin, anemia in IRIDA patients is resistant to oral iron administration and difficult to treat even with parenteral iron, currently the only therapeutic option available.

### Anemia of Inflammation

Chronic inflammatory disorders, including infections, rheumatologic disorders, and inflammatory bowel disease, are commonly affected by hypoferremia and anemia. The anemia is classically mild to moderate and normocytic, but sometimes microcytic and hypochromic, and is hyporesponsive to iron therapy (33). Small observational studies indicate that hepcidin is increased in many patients with these disorders (Figure 1c). Unlike in primary disorders of hepcidin overproduction, such as those due to matriptase-2 mutations, hypochromia and microcytosis are seen in only a minority of patients with anemia of inflammation, perhaps because the latter condition is generally less severe and of shorter duration, and may have a fluctuating course. Individual disease states reflect other effects of inflammation, including shortened erythrocyte survival due to macrophage activation or opsonization of erythrocytes, erythropoietic suppression due to the direct effect of cytokines on erythrocyte precursors, and partial inhibition of erythropoietin production (49). Current treatment of anemia of inflammation is directed at the underlying disease, but this is not always effective. Experimental treatments with different hepcidin antagonists improved anemia in several animal models of anemia of inflammation, but the increasing effectiveness of established and novel anti-inflammatory therapies targeting the underlying disease processes may make human trials challenging.

### Anemia of Chronic Kidney Disease

Anemia of CKD results from a combination of relatively decreased erythropoietin production and the effects of inflammation on iron and erythropoiesis. Erythropoietin levels are actually increased in most patients with anemia of CKD, but this increase may not be adequate for the severity of anemia. Correcting erythropoietin deficiency is not sufficient, as many patients with CKD require high doses of erythropoiesis-stimulating agents to maintain acceptable hemoglobin concentrations. Hyperresponsiveness to erythropoietin is mostly commonly related to iron restriction (50). CKD patients have high circulating hepcidin levels, likely due to the decreased renal clearance as well as the higher production of the peptide. Hepcidin may be induced by inflammation related to the underlying disease process or hemodialysis. The administration of high doses of parenteral iron partially overcomes hepcidin block and facilitates response to erythropoiesis-stimulating agents even in patients with apparently adequate iron stores as indicated by serum ferritin (51). The important role of inflammation in the pathogenesis of CKD anemia was demonstrated by the beneficial effects of anti-IL-6 therapy on anemia in hemodialysis-dependent patients, several of whom were able to discontinue erythropoiesis-stimulating agents altogether (52).

### Anemia of Cancer

Anemia accompanies some malignancies at diagnosis but becomes much more common as the disease progresses, or as a result of chemotherapy and radiation. Depending on the specifics of the disease process, blood loss, malnutrition, infiltration of erythropoietic bone marrow by tumor, and cytotoxic injury to the erythropoietic precursors may contribute to anemia (53). The anemia of some malignancies, including Hodgkin’s disease, multiple myeloma, and myelofibrosis (54–56), resembles anemia of inflammation and is accompanied by increased hepcidin production stimulated by inflammatory cytokines.

### Ferroportin Disease

Ferroportin disease is caused by autosomal dominant missense mutations in ferroportin that lead to decreased synthesis or impaired trafficking of ferroportin, or impaired iron transport function (57). As a result, iron accumulates predominantly in macrophages, leading to severely increased serum ferritin with normal or even low plasma transferrin saturations. Unless exacerbated by factors such as alcoholic liver disease or hepatitis C, the disorder is often clinically silent. Treatment of ferroportin disease with phlebotomy can lead to anemia, revealing the iron-restrictive component of this disorder.

## DIAGNOSTIC USES OF HEPCIDIN

### Hepcidin Assays

Two types of hepcidin assays have been developed for research use: (*a*) immunoassays based on antihepcidin antibodies, which use a reference standard of synthetic hepcidin, and (*b*) mass spectrometric assays, which detect the characteristic mass of the active 25-amino-acid hepcidin species and quantify the intensity of the peak relative to a spiked internal standard (58). Despite discrepancies in the absolute values and normal ranges measured by the various assays, they generally correlate very well. The differences between assays are probably due to the tendency of hepcidin to aggregate and adhere to surfaces, affecting the peptide standards. In healthy subjects, hepcidin levels display diurnal variations manifested by lower morning and higher afternoon hepcidin concentrations (59), as well as a gender difference, with lower hepcidin concentrations in women than in men (60). In pathological conditions, very low hepcidin levels were detected in iron deficiency, β-thalassemia intermedia, and juvenile forms of hereditary hemochromatosis, with somewhat higher levels detected in adult forms of hereditary hemochromatosis (Table 1) (39, 60, 61). Patients with secondary iron overload from transfusions have high hepcidin values unless they have exuberant ineffective erythropoiesis, as is the case in some patients with β-thalassemia major (43). Patients with IRIDA have high-normal or above-normal hepcidin values, despite often severe iron deficiency (46). Inflammatory disorders, multiple myeloma, Hodgkin’s disease, and many cancers also show increased serum hepcidin values (54, 55). Hepcidin is markedly elevated in infections, where it may play a role in host defense by decreasing circulating iron concentrations and limiting the availability of this essential nutrient to microbes (9). Hepcidin concentrations are increased in patients with CKD and correlate with the severity of renal impairment (62).

Clinical hepcidin assays may be useful in the diagnosis of IRIDA, as high-normal or increased serum hepcidin in the presence of iron deficiency is a hallmark of the disorder (46). Other potential applications include the differential diagnosis of hypoferremic anemias due to inflammation versus iron deficiency (63), where low hepcidin concentrations predict therapeutic response to oral iron administration (64). Hepcidin measurements were also shown to be predictive of acute kidney injury after cardiac surgery or in critical illness, of mortality in patients with severe acute kidney injury, and of mortality in septic patients in intensive care (65–70). Standardization of hepcidin assays will be necessary for broad implementation of hepcidin measurements and for testing of additional indications that may benefit from measurement of hepcidin (71).

## HEPCIDIN-BASED THERAPEUTICS

### Hepcidin Agonists

Treatment with hepcidin agonists would be expected to prevent or alleviate iron overload in hepcidin deficiency diseases, including most forms of (hereditary) hemochromatosis and β-thalassemia. In principle, hepcidin can be augmented by stimulating endogenous hepcidin production (e.g., by inactivating TMPRSS6) or by administering exogenous hepcidin or hepcidin agonists. Any of these approaches would be expected to reverse the hyperabsorption and maldistribution of iron. Indeed, multiple hepcidin mimetics have been shown to prevent iron overload in mouse models of hemochromatosis, improve anemia in a mouse model of thalassemia intermedia, and control polycythemia in a mouse model of polycythemia vera (72–74). A hepcidin agonist peptide, rusfertide, is in human clinical trials for the treatment of polycythemia vera, where it appears to effectively replace phlebotomy without exacerbating symptomatic iron deficiency (75). Other hepcidin agonists in clinical trials include the small molecule VIT-2763 (76), as well as antisense oligonucleotides and siRNAs targeting TMPRSS6/matriptase-2, which increase endogenous hepcidin production (74, 77).

### Hepcidin Antagonists

Antagonists not only should be useful in the treatment of iron-restrictive anemias with high circulating hepcidin concentrations but also could release iron from stores and potentiate the effect of erythropoiesis-stimulating agents even when hepcidin concentrations are within the normal range. Four classes of such agents are envisioned (78, 79).

Class I agents inhibit hepcidin production. For example, they may target the stimulatory cytokines IL-6 or BMP6 and associated pathways. Promising approaches include anti-IL-6 antibodies and anti-IL-6 receptor antibodies, already approved for the treatment of rheumatoid arthritis and Castleman’s disease (80). A clinical trial of anti-IL-6 showed remarkable efficacy in anemia of CKD (52). Whether the toxicity from their immunosuppressive effects would prevent more general use for the treatment of anemia, in settings other than those already approved, remains to be seen. The BMP pathway may also be targeted by monoclonal antibodies against hemojuvelin, by soluble hemojuvelin (which acts as an antagonist of BMP signaling), or by recombinant erythroferrone, all of which have been shown to lower hepcidin levels and are currently in preclinical development (81–83). Small-molecule inhibitors of the kinase activity of BMP type I receptors may also be therapeutically useful but would have to be relatively specific for the hepcidin-regulatory pathway (56).

Hepcidin antagonists of class II sequester or inactivate the hepcidin molecule. Hepcidin-neutralizing monoclonal antibodies have been reported by research groups at both Lilly and Amgen (84, 85). The latter antibody increased hemoglobin when combined with ineffective amounts of erythropoiesis-stimulating agents, or alone at higher doses, in a mouse model of anemia of inflammation. Class III agents include monoclonal antibodies that block the hepcidin-binding site of ferroportin and have shown modest activity in anemia of CKD (86). Class IV agents that inhibit ferroportin internalization are also being developed, but their specificity and in vivo effectiveness have not been reported.

## Figures and Tables

**Table 1:** Hepcidin regulation and dysregulation in physiological and pathological conditions

Causation	Disease/condition	Pathophysiology	Serum/plasma hepcidin
Hepcidin changes alter iron homeostasis	Iron-refractory iron deficiency anemia (*TMPRSS6* mutations)	Genetic overproduction of hepcidin causes iron restriction	High normal to high
	Infections, rheumatologic diseases, inflammatory bowel disease, cancer	Inflammation increases hepcidin synthesis, resulting in iron restriction	High
	Chronic kidney disease	Inflammation and decreased renal clearance of hepcidin results in hepcidin excess and iron restriction; iron therapy may raise hepcidin further	High
	Hereditary hemochromatosis (*HFE*, *TFR2*, *HJV*, *HAMP* mutations)	Genetic hepcidin deficiency causes iron overload	Undetectable, low, or low for iron load
	Non-transfusion-dependent thalassemia	Ineffective erythropoiesis and high erythropoietic drive stimulate erythroferrone production, suppressing hepcidin and resulting in iron overload	Low
	Hepatitis C, alcoholic liver disease	Suppression of hepcidin by alcohol, virus, growth factors, and loss of hepatocytes results in iron loading	Low
	Pregnancy	Pregnancy-related hepcidin-suppressive factor results in iron mobilization	Low
Hepcidin changes appropriately reflect iron physiology	Iron deficiency	Blood loss, malnutrition	Low/undetectable
	Secondary iron overload	Transfusions, iron therapy	High
	Hereditary hemochromatosis (*SLC40A1* mutations)	Ferroportin resistance to hepcidin causes iron overload	High
Mixed disorders	Transfusion-dependent thalassemia	Ineffective erythropoiesis suppresses hepcidin, and transfusional iron overload increases hepcidin	Relatively low for iron load, fluctuates during the transfusion cycle

## SUMMARY POINTS

1. The peptide hormone hepcidin regulates the absorption of dietary iron and systemic iron distribution (Figure 1a). Apart from maintaining iron homeostasis, hepcidin controls iron availability in stress conditions, including during increased erythropoietic activity (Figure 1b), during pregnancy, and as part of the host defense during infections (Figure 1c).
2. Pathological overproduction of hepcidin contributes to iron-restrictive disorders (Figure 1c), including anemia of inflammation, iron-refractory iron deficiency anemia, and anemia of chronic kidney disease.
3. Hemochromatosis is caused by hepcidin deficiency due to autosomal recessive mutations in the hepcidin gene or its regulators (Figure 1d), or by rare autosomal dominant ferroportin mutations that confer resistance to hepcidin.
4. Hepcidin suppression by erythroferrone and possibly by other erythroid suppressors of hepcidin contributes to iron overload in iron-loading anemias, including β-thalassemias (Figure 1d).
5. Measurements of serum hepcidin may be useful in the differential diagnosis of iron deficiency and the prediction of responses to oral iron therapy.
6. The hepcidin–ferroportin axis can be targeted for agonists and antagonists to treat iron disorders and anemia.

## FUTURE ISSUES

1. Hepcidin regulation: What are the cellular, biochemical, and structural mechanisms that sense hepatic and plasma iron concentrations and transduce them into transcriptional regulation of hepcidin synthesis?
2. Ferroportin: How does ferroportin transport iron?
3. Pathophysiology: What are the mechanisms that mediate hepcidin suppression during pregnancy? Are there additional suppressors of hepcidin during stress erythropoiesis or in anemias of ineffective erythropoiesis?
4. What are the roles of extrahepatic hepcidin in different tissues and organs and under different pathophysiological conditions?
5. Diagnostics and therapeutics: For which specific diagnostic indications are hepcidin assays superior to current diagnostics? Which diseases and conditions will benefit from treatment with hepcidin agonists and antagonists?
